# Endoplasmic Reticulum Stress and Homeostasis in Reproductive Physiology and Pathology

**DOI:** 10.3390/ijms18040792

**Published:** 2017-04-08

**Authors:** Elif Guzel, Sefa Arlier, Ozlem Guzeloglu-Kayisli, Mehmet Selcuk Tabak, Tugba Ekiz, Nihan Semerci, Kellie Larsen, Frederick Schatz, Charles Joseph Lockwood, Umit Ali Kayisli

**Affiliations:** 1 Department of Histology & Embryology, Cerrahpasa Medical Faculty, Istanbul University, Istanbul 34098, Turkey; tugba.ekiz@istanbul.edu.tr; 2 Department of Obstetrics & Gynecology, Morsani College of Medicine, University of South Florida, Tampa, FL 33612, USA; sefaarlier@gmail.com (S.A.); ozlem2@health.usf.edu (O.G.-K.); nsemerci@mail.usf.edu (N.S.); klarsen@health.usf.edu (K.L.); fschatz@health.usf.edu (F.S.); cjlockwood@health.usf.edu (C.J.L.); 3 Department of Obstetrics & Gynecology, Adana Numune Training and Research Hospital, Adana 01370, Turkey; 4 Department of Obstetrics & Gynecology, Adiyaman University School of Medicine, Adiyaman 02100, Turkey; drselcuktabak@gmail.com

**Keywords:** endoplasmic reticulum stress, decidua, uterus, placenta, ovary, testes

## Abstract

The endoplasmic reticulum (ER), comprises 60% of the total cell membrane and interacts directly or indirectly with several cell organelles i.e., Golgi bodies, mitochondria and proteasomes. The ER is usually associated with large numbers of attached ribosomes. During evolution, ER developed as the specific cellular site of synthesis, folding, modification and trafficking of secretory and cell-surface proteins. The ER is also the major intracellular calcium storage compartment that maintains cellular calcium homeostasis. During the production of functionally effective proteins, several ER-specific molecular steps sense quantity and quality of synthesized proteins as well as proper folding into their native structures. During this process, excess accumulation of unfolded/misfolded proteins in the ER lumen results in ER stress, the homeostatic coping mechanism that activates an ER-specific adaptation program, (the unfolded protein response; UPR) to increase ER-associated degradation of structurally and/or functionally defective proteins, thus sustaining ER homeostasis. Impaired ER homeostasis results in aberrant cellular responses, contributing to the pathogenesis of various diseases. Both female and male reproductive tissues undergo highly dynamic cellular, molecular and genetic changes such as oogenesis and spermatogenesis starting in prenatal life, mainly controlled by sex-steroids but also cytokines and growth factors throughout reproductive life. These reproductive changes require ER to provide extensive protein synthesis, folding, maturation and then their trafficking to appropriate cellular location as well as destroying unfolded/misfolded proteins via activating ER-associated degradation mediated proteasomes. Many studies have now shown roles for ER stress/UPR signaling cascades in the endometrial menstrual cycle, ovarian folliculogenesis and oocyte maturation, spermatogenesis, fertilization, pre-implantation embryo development and pregnancy and parturition. Conversely, the contribution of impaired ER homeostasis by severe/prolong ER stress-mediated UPR signaling pathways to several reproductive tissue pathologies including endometriosis, cancers, recurrent pregnancy loss and pregnancy complications associated with pre-term birth have been reported. This review focuses on ER stress and UPR signaling mechanisms, and their potential roles in female and male reproductive physiopathology involving in menstrual cycle changes, gametogenesis, preimplantation embryo development, implantation and placentation, labor, endometriosis, pregnancy complications and preterm birth as well as reproductive system tumorigenesis.

## 1. Introduction

The cytoplasm of eukaryotic cells contains an endoplasmic reticulum (ER) consisting of a network of flattened sacs and a labyrinth of branching membranous tubules [1] continuous with the nuclear membrane. The initial observations of ER structure were made by Porter et al. [1] and the term “ER” was first coined by Porter and Fullman in 1952 [2]. Development of the ER is postulated to originate either by budding off of the nuclear envelope or by invagination of the plasma membrane [3,4]. Comprising between 15%–60% of the total cell membrane volume, the ER is an evolutionarily specified cellular organelle controlling synthesis, folding, modification, and trafficking of secretory and cell-surface proteins and, generally contains large numbers of attached ribosomes (granular or rough ER). By serving as the major intracellular calcium (Ca^2+^) storage compartment, the ER plays a critical role in maintaining Ca^2+^ homeostasis among various cellular organelles [5,6]. The ER also makes an essential contribution to cell structure/functions by producing the majority of membrane lipids required by other organelles [7,8,9].

Proteins are synthesized as simple linear polypeptide chains followed by complex glycosylation and folding before assuming their final structural and functional conformation. One-third of all proteins synthesized in the ER include secretory proteins that contribute to extracellular structures and functions, plasma membrane proteins that bridge the intracellular and the extracellular milieu, and ER luminal proteins that modulate ER functions. Compared with the cytoplasm, the ER lumen displays a much higher oxidative capacity and contains higher Ca^2+^ levels. This extra-oxidative and hyper-calcemic ER milieu plays a crucial role during protein synthesis and folding [6,10] since protein folding is an energy-dependent process and incorrect folding/glycosylation may occur in the presence of glucose deprivation [11].

In response to extracellular and/or intracellular modifications, cells maintain physiologic functions by mediating a balance between protein synthesis and degradation via a mechanism termed protein homeostasis. Protein synthesis in the ER involves several molecular “inspectors” that assess the quantity and quality of synthesized proteins as well as folding into their native structure [12]. Excess accumulation of unfolded/misfolded proteins in the ER lumen activates an ER-specific adaptation program, (the unfolded protein response; UPR) that increases ER-associated degradation (ERAD) of these structurally abnormal and functionally impaired proteins to maintain protein homeostasis. Several reports provide strong evidence that disruption of protein homeostasis (which includes impaired synthesis, increased unfolding or misfolding and/or excess/reduced degradation) in the ER results in aberrant cellular responses, contributing to the pathogenesis of various diseases [13,14,15,16,17]. This review focuses on ER stress, a homeostatic coping mechanism, particularly the UPR, and its potential impacts in reproductive physiology and pathology that includes menstrual cycle changes, gametogenesis, preimplantation embryo development, implantation and placentation, labor, endometriosis, pregnancy complications and preterm birth as well as reproductive system tumorigenesis.

## 2. The Endoplasmic Reticulum (ER) Stress Induced Unfolded Protein Response (UPR) Signaling Cascades are Vital to Sustain ER Homeostasis

**Figure 1:**
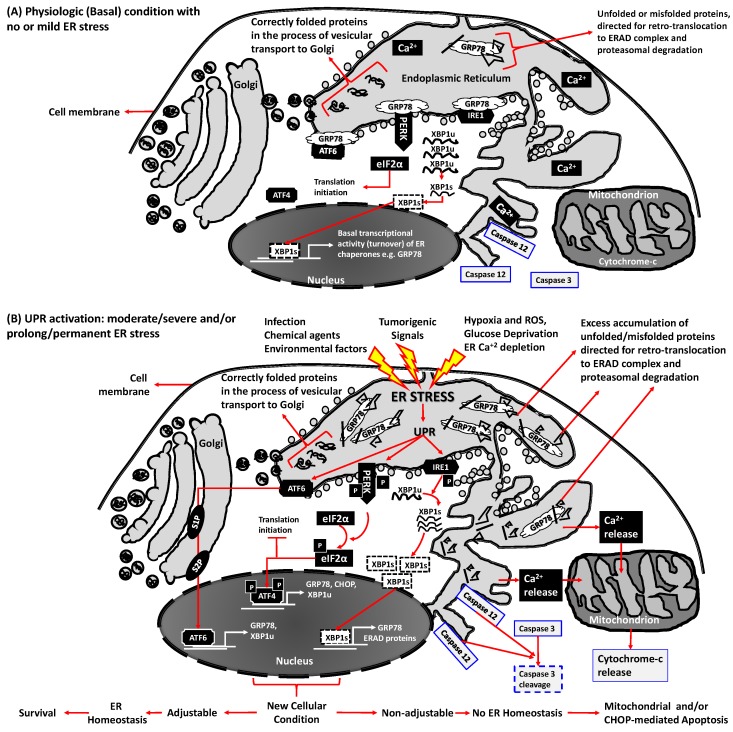
Endoplasmic Reticulum (ER) homeostasis/stress and the unfolded protein response (UPR) signaling in physiopathologic conditions. The UPR consists of three signaling pathways initiated by detachment of upstream transducers activating transcription factor 6 (ATF6), protein kinase R (PKR)-like endoplasmic reticulum kinase (PERK) and inositol-requiring enzyme 1 (IRE1) from glucose-regulated protein 78 (GRP78), a chaperone protein that monitors accumulation of unfolded and misfolded proteins inside the ER lumen. (**A**) In physiological (unstressed) states, these transducers bind to the folding chaperone GRP78 and keep the ER quiescent; (**B**) ER stress inducers accumulate unfolded/misfolded proteins in the ER lumen by impairing protein folding. Higher GRP78 affinity for unfolded/misfolded proteins dissociates GRP78 from ATF6, PERK and IRE1, enabling GRP78 unfolded/misfolded protein binding that then initiates three UPR signaling cascades. Specifically: (1) ATF6 signaling involves its translocation to Golgi apparatus for proteolytic cleavage by site-1 protease (S1P) and site-2 protease (S2P) and subsequent release into the nucleus as an active transcription factor to induce expression of GRP78, ubiquitously expressed X-box binding protein 1 (XBP1u) etc.; (2) PERK signaling consist of auto-phosphorylation of PERK (P-PERK), generating an active kinase that phosphorylates eukaryotic translation-initiation factor 2α (P-eIF2α). P-eIF2α blocks its translation initiating activity and induces ATF4 phosphorylation (P-ATF4) leading to P-ATF4 nuclear translocation as a transcription factor to induce expression of GRP78, C/EBP homologous protein (CHOP), XBPu etc.; (3) IRE1α signaling includes IRE1α phosphorylation (P-IRE1α), an active endonuclease that cleaves XBP-1u mRNA to XBP-1s mRNA, which is then translated to an active transcription factor to induce UPR target genes encoding GRP78, ERAD proteins etc. Thus, by increasing ER chaperone protein levels and blocking of eIF2α-mediated protein synthesis, UPR signaling adjusts cells to increased ER stress conditions by transporting excess unfolded/misfolded proteins to ERAD-complex for proteasome-mediated degradation, thereby re-establishing ER homeostasis and sustaining cell survival, whereas prolonged and/or severe ER stress induces apoptosis by CHOP activation, ER-linked caspase 12-mediated caspase 3 cleavage and/or ER Ca^2+^ efflux associated mitochondrial cytochrome-c release.

ER homeostasis is sustained by ER chaperone proteins that include glucose-regulated protein 78 (GRP78), GRP94, calreticulin (CRT) and protein disulfide isomerase (PDI) [18,19,20]. Among these chaperones, GRP78 (immunoglobulin heavy chain-binding protein (BiP)), is a well-characterized member of heat shock 70 kDa (HSP70) protein family encoded by the heat shock 70 protein 5 (*HSPA5*) gene [21,22]. Residing within the ER as a Ca^2+^-dependent molecular chaperone, GRP78 plays crucial roles in facilitating proper protein folding, maintaining proteins in a folded state, preventing aggregation of protein folding intermediates and directing unfolded and/or misfolded proteins to ERAD (Figure 1A) [18,23,24,25]. Another important function of GRP78 is Ca^2+^ storage within the ER lumen that sustains intracellular Ca^2+^ homeostasis [19]. Since intracellular Ca^2+^ signals contribute to modulation (activation and/or inhibition) of several cellular events, GRP78 regulation of intracellular Ca^2+^ levels is involved in a wide variety of cellular processes including mitochondrial function, cell membrane Ca^2+^ channels, cytosolic Ca^2+^/calmodulin signaling, etc. In addition, GRP78 may play a protective role in cell survival under specific cellular stress conditions by forming complexes with pro-caspases such as caspase-7 and caspase-12 in the ER membrane (Figure 1A). Through these various interactions, GRP78 likely regulates the balance between cell survival and apoptosis in ER-stressed cells [18,26]. Moreover, GRP78 is required during early embryonic development and its expression is decreased during aging [27,28]. The physiological production of exportable proteins is sufficient to induce the synthesis of GRP78 [25]. The requirement for additional resident ER proteins is increased in parallel with an elevation in the secretory workload [29].

Several studies localized GRP78 to the plasma membrane of various cell types where it may function as a surface signaling receptor [30,31,32,33]. A receptor-specific induction of GRP78 expression within endometrial glands has been suggested [26]. Although there is evidence of GRP78 secretion by different human and rat cell types [32,34,35,36], the roles of extracellular GRP78 are unclear and under investigation. Overloading of ER with proteins, as well as hypoxia, oxidative stress, impaired Ca^2+^ homeostasis and glucose deprivation all lead to the accumulation of misfolded or unfolded proteins that induce ER stress resulting in activation of UPR, an evolutionarily conserved mechanism, to cope with the stress condition [37] (Figure 1). A previous study showed increased uterine epithelial GRP78 expression in rats specifically on Day 5 of pseudo-pregnancy and suggested that this time-sensitive increase in GRP78 expression may specifically be required for the efficient biosynthesis and secretion of proteins involved in the onset of uterine sensitization for decidual reaction [26]. Likewise, Beaton et al. [38] observed an increase in GRP78 expression in primary mouse mammary epithelial cells in response to lactation and in the mouse mammary epithelial cell line COMMAD stimulated with prolactin suggesting that induction of GRP78 may function in the processing and secretion of milk proteins. Thus, during both lactation and uterine sensitization for decidualization, GRP78 may function as a crucial regulator of a restricted set of proteins required for secretion. Moreover, in addition to these stress-associated conditions, GRP78 has also been shown to be regulated by specific growth factors including colony stimulating factor-1, erythropoietin, interleukin-3 (IL-3) through a pathway unrelated to stress induction [39].

## 3. ER Stress, UPR Signaling and ER Homeostasis

Various physiological conditions associated with increased protein demand result in enhanced levels of unfolded and/or misfolded proteins that accumulate in the ER lumen. These increases induce ER stress, which triggers activation of UPR signaling [40,41]. The UPR signaling pathway links the ER lumen with the cytoplasm and nucleus, thus enhancing the capacity of a cell to cope with stress. In highly specialized secretory cells such as plasma cells and pancreatic β-cells, the ER compartment expands considerably to compensate for the high volume of protein trafficking. In these cells, the ER may experience accumulation of partially folded proteins that require chaperone assistance.

Impaired protein folding, as exemplified by increased mal-folded protein accumulation, is associated with neurodegenerative disorders such as Alzheimer’s and Parkinson’s disease, as well as prion protein diseases [42,43,44,45]. Furthermore, induction of GRP78 in multiple types of solid tumors is attributed to glucose starvation resulting from poor perfusion within tumors as well as hyper-metabolic characteristics of cancer cells that require much higher glucose utilization rates [18].

As depicted in the scheme in Figure 1A, in non-stressed cells, GRP78 binds to the luminal domain of three ER resident membrane proteins (UPR signal transducers): Inositol-requiring enzyme 1 (IRE1 encoded by endoplasmic reticulum to nucleus signaling 1 gene, *ERN1*), protein kinase R (PKR)-like endoplasmic reticulum kinase (PERK encoded by the eukaryotic translation initiation factor 2-α (eIF2α) kinase 3 gene, *EIF2AK3*), and activating transcription factor 6 (ATF6). Due to the higher binding affinity of GRP78 for unfolded/misfolded proteins, ER stress induced excess unfolded/misfolded proteins promote dissociations between GRP78 and UPR signal transducers ATF6, PERK and IRE1α (Figure 1B). The resulting dissociations ameliorates the accumulation of unfolded/misfolded proteins in the ER by their binding to GRP78, which subsequently retro-translocate them to ERAD complex and proteasome-mediated degradation, resulting in protection of the cell against ER stress-induced cellular dysfunction and apoptosis [18,37,46].

Concurrently, ATF6, PERK and IRE1α, upon dissociated from GRP78, initiate following three UPR signaling cascades: (1) ATF6 is transferred to Golgi apparatus where it is cleaved by site-1 protease (S1P) and site-2 protease (S2P) and then translocates to the nucleus as activated transcription factor (aka ATF6N) [47]; (2) PERK reduces translational initiation and decreases global protein synthesis by inactivating eIF2α via increasing its phosphorylation levels. This PERK phosphorylated eIF2α also activates ATF4, which is another transcription factor of UPR mediated gene expression [48]. These UPR signal transduction steps include translational attenuation that arrests the entry of new proteins into the ER, transcriptional activation of genes encoding ER chaperone proteins, e.g., GRP78 and GRP94, and ER enzymes e.g., PDI and peptidyl-prolyl isomerase involved in protein folding that assists in the maturation of proteins, and transcriptional activation of genes functioning in the ERAD system to decrease the number of misfolded proteins by proteasome mediated degradation as well as pro-apoptotic protein C/EBP homologous protein (CHOP) (Figure 1) [49,50,51]; (3) IRE1 activates X-box binding protein 1 (XBP1, a transcription factor regulating UPR-associated genes) -mediated signaling [52,53]. Specifically, as a result of detachment from GRP78, IRE1α is released to the cytoplasm and becomes an active endonuclease that catalyzes the excision of a 26 nucleotide unconventional intron from ubiquitously expressed XBP1 (XBP1u) mRNA resulting in a translational frame shift that produces a 371 amino acid isoform, XBP1 spliced (XBP1s) (Figure 1B). Consequently, XBP1s acts as a transcription factor that enhances the expression of ER chaperones, specifically GRP78 levels, and therefore maintains ER homeostasis [18,37,46]. IRE1α and XBP1s can also interact with the phosphatidylinositol-4,5-bisphosphate 3-kinase(PI3K) regulatory subunits, p85α or p85β and IκB kinase β (IKKβ) that may modulate relevant intracellular signaling [54,55]. However, if the primary stimulus that causes ER stress is either prolonged or severe, cell death primarily by apoptosis is induced [56,57,58], specifically by coupling of the ER with mitochondrial pathways [50] suggesting that the UPR protects cells from mild stress, but can also initiate apoptosis if ER stress inducers are sustained and become intolerable (Figure 1B).

## 4. UPR Signaling, ER Stress in Reproductive Physiopathology

### 4.1. The Menstrual Cycle Endometrium

The human endometrium is a dynamic tissue that displays spatial and temporal changes in proliferation, apoptosis, angiogenesis, decidualization as well as extracellular matrix remodeling to control tissue differentiation and growth under the influence of the sex steroids estrogen and progesterone as well as numerous local paracrine and autocrine factors during the menstrual cycle [59,60]. Estradiol (E_2_) exerts its classical (genomic) effects by binding to E_2_ receptors ERS1 and ESR2 (aka ERα and ERβ, respectively) to regulate transcription [61] and/or by non-classical (non-genomic) mechanisms by binding to receptors located at the cell membrane, mitochondria, and the ER [62,63].

**Figure 2:**
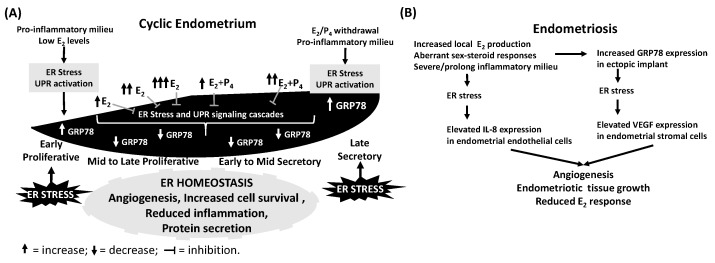
Endometrial regulation and role of ER stress during menstrual cycle and in endometriosis. Human endometrium undergoes several cellular, molecular and morphologic changes during menstrual cycle. (**A**) In situ studies demonstrate increased GRP78 expression during late secretory and early proliferative phases, which may be result from low estradiol (E_2_) levels and/or pro-inflammatory milieu. In culture, E_2_ blocks ER stress (tunicamycin)-induced GRP78 expression supporting a role for E_2_ in favor of ER homeostasis by suppressing GRP78 (ER stress sensor) during the E_2_ dominated phases of the cycle, which directly or indirectly contributes to angiogenesis, cell proliferation/apoptosis and protein secretion occurring each cycle; (**B**) Alternatively, significantly high GRP78 levels in ectopic endometriotic tissues may result from increased local E_2_ production, aberrant sex steroid signaling (progesterone resistance) and/or an enhanced increased inflammatory milieus. These severe/prolonged conditions may activate ER stress/UPR signaling cascades to enhance VEGF expression and induce angiogenesis, required for endometriotic tissue growth. Moreover, significant reduction in estrogenic response in severe ER stress (tunicamycin) condition may contribute to aberrant steroid response in endometriotic tissues.

Several immunohistochemical studies revealed menstrual cycle-dependent changes in the levels of various members of the heat shock family of proteins in human endometrium, as exemplified by HSP70, and observed steroid hormone regulation of HSPs and reported an association between down-regulation of both the estrogen and progesterone receptors and overexpression of HSP70 in secretory endometrial glands [64,65]. As a member of the HSP70 protein family, GRP78 expression is tightly regulated at the site of embryo implantation in mice [66]. However, few studies have evaluated the regulation and potential roles of GRP78 during cyclic changes of human endometrium. Our prior studies identified cycle-dependent alterations in GRP78 expression in human endometrium and revealed an inverse correlation between GRP78 expression and E_2_ levels [67]. This in situ regulation of GRP78 levels in human endometrium differs from regulation of the HSP70 protein, which displays a strong increase in human endometrium during late proliferative and early secretory phases in a topological manner (higher in basalis vs. functionalis endometria) [64,65]. E_2_ signaling may directly regulate GRP78 levels and thus contribute to ER homeostasis, or indirectly impact on GRP78 expression as a result of peri-menstrual E_2_ withdrawal to elicit increased endometrial recruitment of leukocytes that produce high levels of inflammatory cytokines and reactive oxygen species (ROS) (Figure 2). Therefore, the ability of both pro-inflammatory cytokines and ROS to induce HSP70 expression [68] may activate ER stress and UPR signaling in human endometrium during the late secretory and early proliferative phases [69,70,71,72]. These changes may result in stimulation of GRP78 expression through activation of XBP1-mediated UPR signaling [53,73].

Although the specific function(s) of GRP78 in human endometrium is/are unclear, the presence of GRP78 expression in both glandular and stromal cells throughout the menstrual cycle supports its involvement in regulating changes in the endometrium by controlling protein folding, intracellular Ca^2+^ balancing and degradation of unfolded and/or misfolded proteins. These actions, in turn, prevent activation of the UPR signaling cascades and cellular stress, and may participate in the maintenance of endometrial tissue growth and homeostasis [67]. In the mouse uterus, GRP78 is induced by E_2_ via an estrogen receptor-independent mechanism [74]. However, the expression of GRP78 is tightly regulated at the site of embryo implantation, an event that is regulated by E_2_ [66]. Moreover, in vitro experiments with mouse uterine stromal cells show that GRP78 levels are not expressed in the absence of E_2_, but are upregulated within 2 h and remain unchanged for 24 h following E_2_ incubation [75]. By comparison, we observed no changes in GRP78 expression following 8 and 24 h of E_2_ treatment in both human endometrial stromal and glandular cell cultures [67,76] indicating that our in situ findings likely reflect indirect E_2_ effects. Moreover, our in vitro experiments also revealed significantly decreased tunicamycin (a strong ER stress inducer)-enhanced GRP78 expression in the presence of E_2_, which supports our in situ results showing lower levels of GRP78 in stromal and epithelial cells during the mid- and late-proliferative and mid-secretory phases and suggests that an E_2_-mediated mechanism inhibits UPR signaling in the endometrium to maintain tissue growth and homeostasis (Figure 2). Further analysis using the alkaline phosphatase assay, a well-documented technique that measures in vitro estrogenic potency of a compound [77], revealed that tunicamycin-mediated activation of UPR signaling significantly blocks estrogenic responses in Ishikawa cells, a human endometrial adenocarcinoma cell line [67]. This finding is independent of the proliferative and/or anti-proliferative effects of tunicamycin or E_2_ as determined by a cell survival assay. Taken together, these results provide strong evidence of the existence of negative bidirectional cross-talk between E_2_ and UPR signaling pathways [67].

A separate study [78] compared ER homeostasis in human endometrial endothelial cells among menstrual cycle phases and found the highest GRP78 expression in the late secretory phase. Moreover, the pro-inflammatory cytokines, tumor necrosis factor α (TNFα) and IL1β increased GRP78 levels in primary human endometrial endothelial cell cultures, and enhanced IL8 secretion. This study also showed that tauroursodeoxycholic acid (TUDCA, an endogenous bile acid and inhibitor of ER stress) eliminated TNFα, but not IL1β stimulated IL8 secretion, indicating specific regulation of TNFα-mediated signaling by this ER stress modulator. These observations suggest that pro-inflammatory cytokines likely contribute to the menstrual cycle-dependent modulation of ER homeostasis in the endometrial vasculature as well as during angiogenesis.

### 4.2. Endometriosis

Endometriosis, an estrogen dependent inflammatory condition, is a common gynecological disease in reproductive age women that is characterized by the implantation and growth of endometrial tissue outside the uterus. Endometriosis affects 10% of reproductive age women and up to 50% of infertile women [79,80]. Patients with endometriosis often suffer from dysmenorrhea, dyspareunia, dysuria and chronic abdominal or pelvic pain as well as infertility, resulting in a limited quality of life [81]. The pathogenic mechanisms underlying the development of this disorder are not fully understood. Among several proposed hypotheses, retrograde menstruation, i.e., the flow of menstrual debris from the uterus along the fallopian tubes into the pelvis is the most widely accepted [82]. Women susceptible to developing endometriosis likely have an optimal tissue microenvironment for the adhesion and growth of endometrial cells at ectopic sites. Thus, the pathogenesis of endometriosis involves a complex interplay of genetic, anatomical, environmental, and immunological factors [81]. Degradation of the extracellular matrix (ECM) as well as inflammation and angiogenesis are also important processes in the nidation, survival and progression of ectopic endometriotic cells/tissues [83,84]. In addition, a small population of adult/progenitor stem cells with mesenchymal stem cell characteristics are present in endometrial debris reaching the peritoneal cavity during retrograde menstruation. These cells have been recently implicated in generating endometriotic implants by differentiating into endometrial cells via their proliferative, invasive and neo-angiogenic properties.

Recent studies reported involvement of epithelial-mesenchymal transition (EMT)-like and mesenchymal-epithelial transition (MET)-like cellular processes as well as increased oxidative and ER stress mechanisms in the pathogenesis of pelvic endometriosis [67,85,86]. To identify/investigate the role of GRP78 regulation in the pathogenesis of endometriosis, we investigated the expression of GRP78 in normal human endometrial and endometriotic cells in situ. Immunoreactive GRP78 levels in ectopic epithelial cells were significantly higher than in cells of paired-eutopic endometrium, suggesting activation of UPR signaling cascade in ectopic implants [87]. In addition to our in situ findings, Taylor et al. [88] reported that induction of cultured endometrial stromal cells by tunicamycin elevates vascular endothelial growth factor (VEGF) expression, providing evidence for the functional involvement of UPR in angiogenesis in ectopic endometrial implants. This UPR induction of VEGF levels in endometrial stromal cells as well as a TNFα-induced UPR mediated increase in IL-8 expression in human endometrial endothelial cells indicate involvement of ER stress in the pathogenesis of endometriosis by supporting ectopic endometrial angiogenesis, cell survival as well as tissue growth (Figure 2).

### 4.3. Endometrial and Other Reproductive Tissue Cancers

Endometrial cancer is the most common malignancy of the female genital tract. Overall the endometrium is the fourth most frequent cancer site, accounting for 6% of all cancers in women. Risk factors for endometrial cancer include obesity, diabetes, estrogen therapy, polycystic ovarian syndrome and a westernized lifestyle. Immunohistochemical studies by Bifulco et al. [50] revealed that endometrial cancers display increased GRP78, ATF6 and CHOP, a mediator of ER-stress induced apoptosis, mRNA levels and elevated GRP78 and ATF6 protein levels. Another study reported increased plasma membrane localization of GRP78 in endometrial adenocarcinoma tissues [89]. Immunohistochemical analyses of CHOP and p53 expression levels in tissue sections from patients with invasive squamous cell carcinoma or pre-invasive lesions of cervical intraepithelial neoplasia stage I and III observed significantly higher CHOP expression in all of these cancer types compared with control tissues. Moreover, cervical squamous cell carcinomas (invasive) display significantly more frequent CHOP expression compared with cervical intraepithelial neoplasia. CHOP expression is positively correlated with high-risk human papillomavirus infection and p53 expression suggesting existence of an interaction between high-risk human papillomavirus infection and/or p53 signaling and UPR signaling [90]. The effects of p53 on gene expression during the DNA damage response include activation of cyclin-dependent kinase inhibitor 1 (CDKN1A) and Mouse double minute 2 homolog (MDM2) proteins under physiological conditions. However, increased ER-stress and subsequent UPR activation induce an isoform change in the initial 40 amino acids of p53 that results in suppression of CDKN1A and MDM2 protein levels indicating the opposite impact of p53 on expression of the same gene associated with ER-stress levels and/or duration [91]. Taken together, these in situ results provide strong evidence that ER-stress/UPR signaling is involved in reproductive tract tumorigenesis. Indeed, the existence of limited publications regarding in situ studies mandates the need for further studies to investigate cell or tissue-type and stage or grade-dependent changes in expression and activation levels of ER-stress sensors. Moreover, investigations into the functional impact of ER-stress sensors using their over/under-expressed or continuously active forms in relevant animal models of endometrial tumor growth/invasion are required. The results of these studies are expected to improve understanding of the precise contribution of ER-stress adaptation mechanism(s) in the development and progression not only poof reproductive tract but also other cancer types.

Ca^2+^ signaling involved in controlling mitochondria and/or ER mediated apoptosis is crucial in regulating cancer cell survival. Furthermore, by dealing with stressful metabolic environments, altered autophagy mechanisms in cancer cells prevents ER-stress induced apoptosis, which is considered to be an adaptation mechanism during tumorigenic differentiation and growth [92,93]. However, autophagy likely plays dual roles in either providing protection from or inducing cell death [94]. As noted above, the in situ stimulation of GRP78 or CHOP in cancers of reproductive tissues indicates that adaptation of ER stress may be crucial in regulating tumor survival/growth and may be involved in inducing chemotherapy resistance, which suggests that alone or in combination with currently available chemotherapeutics, use of such agents that impair ER-homeostasis and/or disrupt cancer cell ER-stress adaptation mechanism(s) may provide more effective therapies. Indeed, functional studies revealed that the effect of GRP78 knock-down caused a decreased growth rate of Ishikawa cells and increased apoptosis of AN3CA cells (an endometrial adenocarcinoma cell line) in culture [50,89]. Consistent with this hypothesis, paclitaxel induces the expression of Beclin 1 and Microtubule-associated protein 1A/1B-light chain 3 (LC3) II, which are proteins that play central roles in autophagy, suggesting that paclitaxel-induced apoptosis may be mediated by activation of autophagy in cultured HeLa cells (a cervical adenocarcinoma cell line). However, further potentiation of apoptosis in HeLa cells pre-treated with either the autophagy inhibitor chloroquine or small interfering RNA against Beclin 1 found that although paclitaxel induces expression of proteins involved in autophagy, its apoptotic impact is likely mediated by a different mechanism than autophagy displayed in HeLa cells. Additional analysis of this inhibition of autophagy in HeLa cells revealed increased expression of GRP78 and CHOP levels leading to ER stress mediated apoptosis [95]. These observations suggest that paclitaxel-induced autophagy may be a tumor cell adaptation mechanism to degrade excess unfolded or misfolded proteins accumulation, thereby preventing prolong/severe ER stress, which blocks UPR-mediated apoptosis and thus promotes tumor cell survival. Moreover, cisplatin, another chemotherapeutic agent, significantly increases cellular Ca^2+^ concentrations and triggers ER dependent apoptotic cascades by inducing expression of GRP78 and CHOP as well as activation of caspase-4 and autophagy-mediated degradation of ubiquitinated proteins. Taken together, these observations demonstrate that both Ca^2+^ efflux and autophagy induction by ER-stress play significant roles in mediating cisplatin induced apoptosis in cervical adenocarcinoma [96,97].

**Figure 3:**
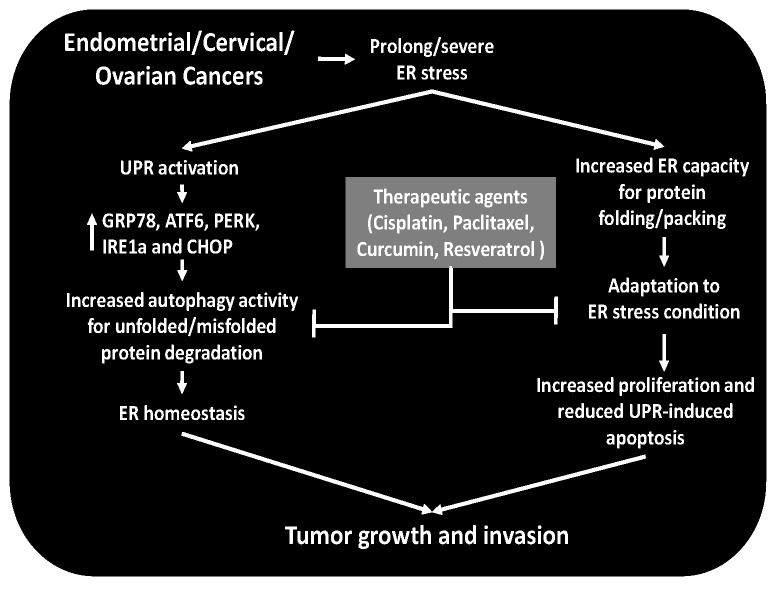
Regulation and therapeutic targeting of ER stress in reproductive tissue cancers. Several reproductive tissue cancers display increased ER stress chaperone GRP78 and the UPR signaling proteins ATF6, PERK and IRE levels in situ, indicating increased ER stress with a cancer cell specific adaptation to the stress condition via increased autophagy activities to degrade unfolded/misfolded protein as well as enhanced ER protein folding capacity, which result in ER homeostasis, thereby supporting growth and invasion of tumor cells. Several studies reported that disruption of cancer cell specific adaption to severe/prolong ER stress by chemotherapeutic agents (cisplatin, paclitaxel) or naturally occurring agents (curcumin, resveratrol etc.) can reduce cancer cell proliferation and invasion and increase apoptosis, resulting in tumor growth regression.

Similarly, curcumin treatment of several cervical cancer cell lines, i.e., C33A, CaSki, HeLa, and ME180, but not normal epithelial cells or peripheral blood mononuclear cells, results in reduced proliferation and elevated apoptosis by activating ER-stress sensors of UPR signaling, e.g., PERK, IRE-1α, ATF6 and CHOP (a key factor involved in ER stress-mediated apoptosis). Moreover, curcumin-induced CHOP expression and ROS generation in these cancer cell types trigger a decrease in the ratio of Bcl-2/Bax proteins (anti-apoptotic/pro-apoptotic proteins, respectively) [98]. Use of RA-9, a small-molecule inhibitor of proteasome-associated deubiquitinating enzymes, causes unsustainable levels of proteotoxic stress and elevates UPR mediated apoptosis in both primary culture of ovarian cancer cells and ovarian cell lines while reducing in vivo tumor growth and increasing overall survival in a manner well-tolerated by the host [76]. Moreover, resveratrol, a natural phenol and phytoalexin produced naturally by grapes, triggers ER stress-mediated apoptosis in ovarian cancer cells via activation of the ER-stress sensors PERK and ATF6α [99]. As briefly schematized in Figure 3, overall, these results from studies noted above support targeting ER stress/UPR mechanisms in cancers as a novel therapeutic approach. However, since these studies are predominantly carried out in vitro, use of these agents alone or in combination with current chemotherapeutics in animal and clinical trials is mandatory to complement the in vitro observations.

### 4.4. Sperm

**Figure 4:**
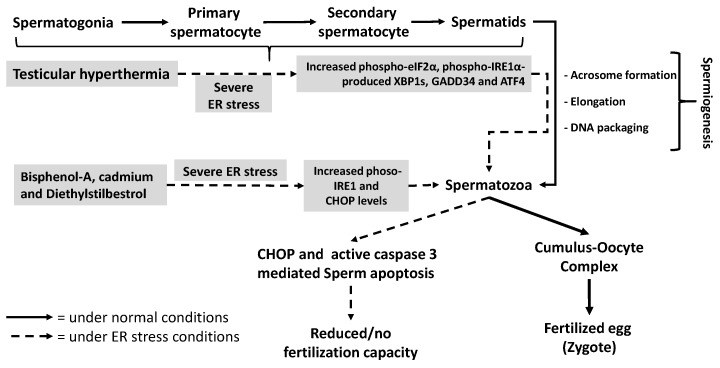
Role of ER stress in spermatogenesis. Increased protein synthesis and/or degradation to compensate intracellular, membranous, biochemical and structural changes during spermatogenesis assign a central role to the ER in coordinating these events. Several reports indicate that testicular hyperthermia induces UPR signaling cascades suggesting that increased ER stress may impair spermatogenesis. Endocrine-disrupting chemicals bisphenol-A and diethylstilbestrol and cadmium, an environmental toxicant, are reported to cause severe ER stress by elevating IRE1α phosphorylation and CHOP expression in spermatozoa. The resulting increased CHOP expression then triggers apoptosis via activating caspase 3 in sperm, which may reduce or eliminate fertilization capacity.

Spermatogenesis requires extensive protein synthesis to differentiate spermatogonia to spermatozoa during mitosis and meiosis within the testes. Moreover, after ejaculation, sperm undergoes sequential intracellular, membranous and biochemical changes during capacitation, in order to fertilize the oocyte in the female genital tract [100,101]. These changes include increases in intracellular Ca^2+^ concentration, pH, cyclic adenosine monophosphate (cAMP), membrane fluidity, and protein tyrosine phosphorylation. Increased tyrosine phosphorylation of sperm proteins is an important aspect of capacitation and has been shown to be associated with hyper-activated motility, zona pellucida binding and acrosome reaction [102,103,104,105]. A study by Lachance et al. [106] evaluated the effects of two recombinant chaperone proteins, Hsp60 and GRP78, on human sperm functions and found that both are expressed by oviduct epithelial cells where they modulate protein tyrosine phosphorylation and intracellular Ca^2+^ levels during spermatozoa capacitation [106]. Two independent studies provide evidence that the toxic effects of endocrine-disrupting chemicals bisphenol-A and diethylstilbestrol and the major occupational and environmental toxicant cadmium on the testis are mediated by impairing ER homeostasis (Figure 4) via induction of IRE1α phosphorylation and CHOP expression in rat spermatozoa [107,108]. By comparison, activation of UPR signaling via enhanced levels of phosphorylated eIF2α, ATF4 and Growth arrest and DNA damage-inducible protein GADD34 (GADD34) and phospho-IRE1α induced XBP1s in response to testicular hyperthermia (43 °C, 15 min/day) as well as elevated ER stress-mediated spermatocyte apoptosis associated with CHOP, phosphorylated-c-Jun NH2-terminal kinases (P-JNK) and caspase-3 activity after repetitive periods of hyperthermia (Figure 4) have all been reported in the mouse testis [109]. In support of these observations, genetically induced excess ER stress as a result of GRP78 knockdown in the drosophila male accessory gland, which secretes seminal fluid proteins essential for reproduction, leads to increased XBP1s levels and results in male infertility [110].

Fertilization takes place in the ampulla region of the oviduct as a result of successful interactions between female and male gametes. Human spermatozoa that have completed capacitation bind to the zona pellucida and release the acrosome content (acrosome exocytosis). On the other hand, acrosome reaction occurs spontaneously [111,112]. Penetration of the zona pellucida by spermatozoa follows perivitelline entry, and is then finalized by binding and fusion to the oocyte plasma membrane [100]. A previous study postulated that GRP78 is expressed and secreted by oviduct epithelial cells and then binds to gametes to modulate epithelium/gamete interaction in a Ca^2+^-dependent manner [113]. Additionally, clinical evidence connecting ER stress to fertilization revealed significantly elevated levels of endometrial GRP78 in women with repeated in vitro fertilization failure compared to those with successful fertilization [114].

### 4.5. Oocytes

**Figure 5:**
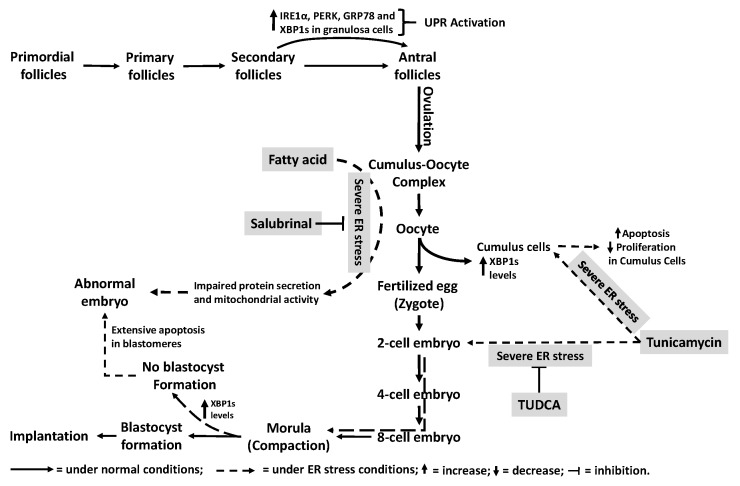
Regulation and the impact of ER stress/UPR signaling cascades during oogenesis and preimplantation. During folliculogenesis, enhanced IRE1α, PERK, GRP78 and XPB1s levels in the granulosa cells of large secondary follicles and later stages as well as elevated XBP1s levels in cumulus cells from fertilized oocytes indicate physiological involvement of UPR signaling during oogenesis and fertilization. However, increased fatty acid levels and obesity can dysregulate protein secretion and mitochondrial activity by impairing ER homeostasis, causing abnormal embryonic development. Salubrinal treatment of cumulus-oocyte complex maintains normal preimplantation embryo development by reversing these conditions. The preimplantation embryo also requires extensive protein synthesis for proper development and implantation. Severe ER stress induced by tunicamycin in 2-cell stage embryos does not affect development until the end of morula stage. However, severe ER stress impairs blastocyst formation via extensive apoptosis. Use of TUDCA completely reverses these negative effects, indicating that ER homeostasis is crucial during blastocyst formation and subsequent development.

Functional protein synthesis occurs via translation of maternal mRNA and is essential for appropriate oocyte development and maturation [115,116,117,118]. During these processes, the ER plays crucial roles to meet increased protein demand by oocytes. This task is accomplished by proper protein synthesis, folding, modification and trafficking. Therefore, regulation of ER homeostasis/stress is likely to be a key mechanism during folliculogenesis and oocyte maturation. Sialic acid-binding lectin (SBL), a major product of bullfrog (*Rana catesbeiana*) oocytes, acts as a strong inducer of ER stress mediated apoptosis by increasing GRP78 levels and activating caspase-4 in Jurkat cells (a human leukemia T-cell line) [119]. In mouse cumulus-oocyte complexes, fatty acid-induced ER stress impairs protein secretion and mitochondrial activity resulting in abnormal embryo development, which is reversed by the ER stress inhibitor salubrinal [120]. Recently, Wu et al. [121] revealed that conception in obese mice imparts a legacy of mitochondrial DNA loss in the offspring that is caused by ER stress. A recent study by Harada et al. [122] investigated the roles of UPR signaling and ER stress in granulosa and cumulus cells during follicular growth and maturation in the mouse ovary. In this study, in situ hybridization and immunohistochemistry analyses revealed expression of both XBP1s and GRP78 as well as activation of ER stress sensor proteins, IRE1 and PERK in granulosa cells at a later stage than large secondary follicles (Figure 5). Furthermore, compared with human cumulus cells isolated from oocytes displaying no sign of fertilization following intra-cytoplasmic sperm injection (ICSI), expression of XBP1s mRNA is two-fold higher in cumulus cells isolated from oocytes exhibiting fertilization (Figure 5). This observation suggests that UPR is required during follicular growth and maturation to obtain normal oocyte development [122]. Similarly, 48 h tunicamycin treatment of human cumulus cells isolated by hyaluronidase removal from MII oocytes during preparation for the ICSI procedure, significantly reduced proliferation and enhanced apoptosis. TUDCA therapy partially, but significantly, reverses this tunicamycin-mediated inhibition of cumulus cell proliferation (Figure 5). This study also observed increased GRP78 protein levels in cumulus cells obtained from women with a poor response to controlled ovarian hyper-stimulation versus those with a normal response (submitted to *Journal of Obstetrics and Gynaecology Research*). A study carried out in mice demonstrated a relationship between obesity and ER stress in cumulus-oocyte complexes associated with reduced mitochondrial membrane potential, high autophagy levels and high intracellular lipid levels. Importantly, pre-ovulatory administration of salubrinal, an ER stress inhibitor (Figure 5), completely restored oocyte quality by increasing levels of the mitochondrial replication factors mitochondrial transcription factor A (TFAM) and dynamin related protein 1 (DRP1) as well as mtDNA in oocytes derived from the obese mice [121]. Overall, these findings suggest that ER homeostasis plays crucial roles in folliculogenesis in the ovary, cumulus cell survival, cumulus-oocyte complex interactions as well as oocyte quality.

### 4.6. Preimplantation Embryo Development

Preimplantation embryos develop under the influence of various hormones and growth factors derived from maternal tissues and/or embryonic sources require transition from maternal to embryonic RNA, followed by extensive protein syntheses [123]. Developing preimplantation embryos synthesize and secrete a wide range of hormones and growth factors that promote embryonic survival and/or subsequent uterine communication to achieve a successful implantation. In the blastocyst stage, a further increase in cell number via activation of transcription as well as protein synthesis [49] may experience an inherent level of ER stress and consequently activate specific coping responses to sustain ER homeostasis and support later stage of embryonic development [49,124]. However, under in vitro conditions, preimplantation embryos are vulnerable to a variety of physicochemical stresses such as shearing, temperature changes, altered pH as well as higher oxygen pressure, all of which are known inducers of ER stress coping responses. These various stressors alter gene expression, epigenetic mechanisms and metabolism that may impair embryonic development and/or viability [125]. In a porcine model, the functional abundance of XBP1s is low in mature oocytes as well as in 1-, 2- and 8-cell stage embryos, but it is highly abundant during the germinal vesicle, 4-cell, morula (compaction) and blastocyst stages [126].

Our research group previously studied the physiopathologic impact of short-term and long-term activation of ER stress in preimplantation embryo development in mice. On day 4 of embryonic development, compared to vehicle (control), tunicamycin-treatment reduced blastocyst formation from 79% to 4% and induced 2-fold and 2.6-fold increase in XBP1 and XBP1s mRNA expression, respectively. These tunicamycin-treated preimplantation embryos also displayed significant nuclear fragmentation (Figure 5). These results suggest that under culture conditions, severe ER stress-mediated activation of UPR signaling contributes to low rates of blastocyst development/formation [127]. During the pre-implantation stage, TUDCA improves maturation and developmental competence of porcine embryos by reducing ER stress-induced apoptosis by interrupting the classic pathways of apoptosis in vitro [128]. Similarly, incubation of mouse embryos with TUDCA improves the rate of two-cell embryo development to blastocysts by attenuating both the expression of XBP1s protein in the nucleus together and ER stress-induced apoptosis [126]. Recent studies showed that addition of TUDCA to culture media, increases both implantation and live birth rates of transferred mouse embryos [129]. Previously, Sharma et al. [130] observed tunicamycin-mediated reduction in the rate of blastocyst development of cultured buffalo embryos, but failed to confirm the reversible impact of TUDCA on this effect, which may reflect prolonged tunicamycin exposure. In this regard, we observed that both the therapeutic and protective effects of TUDCA on tunicamycin-mediated inhibition of blastocyst formation are associated with the induction period of ER stress [127]. Moreover, the IRE1α arm of the UPR pathway is activated in freshly collected embryos, but not in vitrified/thawed embryos [124]. These results suggest that ER stress/homeostasis is essential for preimplantation development and that biotechnologies used in assisted reproduction labs may stress the developing embryo, which activates UPR signaling to copes with these various types of stressors.

### 4.7. Implantation

Previous findings provide evidence that during ER stress and the resulting homeostatic reaction, the UPR plays a crucial role in both the innate and adaptive immune responses [131]. Excessive amounts of cytokines can trigger Ca^2+^ release from the ER and induce ROS generation, leading to ER stress and inflammation and thereby altering the physiological response [131]. Furthermore, ER stress-associated UPR signals are involved in the maintenance of lymphocyte homeostasis and viability [132]. Endometrial GRP78 expression levels are significantly up-regulated in the mid-secretory phase in women with recurrent miscarriage. In these women, this increased GRP78 expression may be associated with an altered immune response during the window of implantation [114]. Moreover, during their preimplantation development, embryos secrete molecules that regulate decidual cell ER functions by inducing expression of heat shock cognate 71 kDa protein (HSC70), a protein responsible for the proper folding of newly translated and misfolded proteins, suggesting preimplantation embryo-mediated paracrine regulation of decidualization that facilitates implantation [133]. Compared with fertile women, increased endometrial levels of GRP78 provide evidence of a link between ER stress and defective implantation [114]. However, more functional studies are required to increase understanding of the precise role of ER stress/homeostasis in early pregnancy.

## 5. Role of ER Stress in Pregnancy Complications and Preterm Birth

Several distinct etiologies contribute to such pregnancy complications as preeclampsia, fetal growth restriction (FGR), gestational diabetes mellitus (GDM) and chorioamnionitis. These conditions are associated with preterm birth (PTB) and remain the leading cause of perinatal morbidity and mortality worldwide. Pathological mechanisms leading to preeclampsia and FGR display considerable overlap [134]. Infections, stress, substance abuse (e.g., smoking), maternal under-nutrition, chromosomal abnormalities, genetic and epigenetic modifications and syndromes with an unknown genetic (inherited) basis are primary mediators of FGR [135]. Each of these factors elicits functional placental insufficiency, a hallmark of FGR [136]. Both FGR and preeclampsia are strongly associated with shallow decidual trophoblast invasion, which leads to incomplete spiral vascular transformation causing insufficient uteroplacental blood flow for the developing maternal-fetal unit. The resulting placental hypoxia/oxidative stress enhances placental secretion of several anti angiogenic factor (e.g., soluble flt-1 and endoglin), which elicit vascular dysfunction/damage leading to maternal hypertension, proteinuria and end-organ dysfunction [137,138,139].

GDM is caused by a progressive decrease in insulin sensitivity and inadequate insulin secretion generally beginning in the late second trimester of pregnancy. Maternal obesity, metabolic dysfunction and genetic susceptibility are strongly associated with GDM. While GDM is major risk factor for macrosomia, it is also a major risk factor for preeclampsia [140,141,142].

Intrauterine infections [143,144] accompany about half of early (<32 weeks) PTBs. Microbial species ascend from the vagina and cervix to the uterus where they initiate deciduitis, then chorioamnionitis, villitis and in extreme cases, fetal infection [144,145]. Amniotic fluid from patients with PTB complicated by intrauterine infections contains elevated levels of TNFα, IL-1β, IL-6 and the primary neutrophil chemoattractant, IL-8 [146,147,148]. In full-thickness amniochorio-decidual membranes, endotoxins and exotoxins induce greater enhancement of IL-1β and TNFα expression when exposure is confined to the decidua but not amnion [149], emphasizing the importance of the decidua in mediating inflammation. In primary human leukocyte-free term decidual cell cultures, IL-1β markedly increases expression of COX-2 [150], IL-6, IL-8 [151] and matrix metalloproteinases (MMP1 and MMP3) [152] suggesting that infection-induced inflammation contributes to PTB by enhancing labor mediators.

Previous studies indicate that ER stress/UPR signaling contributes to pathophysiological regulation of later placental and fetal development stages as well as parturition by affecting functionally available proteins produced by placental cells [153]. Accordingly, significantly higher levels of the ER stress markers, GRP78, P-eIF2α and XBP-1, localized primarily in the syncytiotrophoblast were observed in placentas in-labor versus placentas among patients delivered by cesarean section [154]. Similarly, compared to non-laboring specimens, significantly elevated levels of GRP78, IRE1 and XBP1s are reported in fetal membranes and myometrium during both term and PTB [155]. Moreover, lipopolysaccharide (LPS) treatment stimulates UPR signaling by increasing GRP78, IRE1 and XBP1s in explant cultures of both fetal membranes and myometrium suggesting that bacterial product(s)-mediated disruption of ER homeostasis may contribute to infection-induced PTB [155].

Severe developmental defects in IRE1α exons 7–14 deleted mice cause embryonic lethality after 12.5 days of gestation [156]. Iwawaki et al. [157] showed that IRE1 is activated predominantly in the placenta and that its loss leads to a reduction in VEGF-A levels as well as severe dysfunction of the murine labyrinth placenta, indicating that the IRE1α arm of the UPR coping response is essential for placental development and embryonic viability. Proteomic analysis of placental specimens from early pregnancy loss revealed down-regulation of both GRP78 and valosin-containing protein (VCP), a sensor that detects accumulation of misfolded proteins, specifically in decidual cells at the maternal-fetal interface, suggesting that sustained ER stress acts as a co-factor of oxidative stress and contributes to molecular induction of early pregnancy loss [158]. In association with early pregnancy loss, significantly enhanced GRP78 and ubiquitinated protein levels are detected in cultured decidual cells in response to hydrogen peroxide (H_2_O_2_), thus providing support that excessive ROS production impairs UPR function by decreasing VCP in decidual cells. These changes lead to cell damage, resulting in reduced cell numbers (increase apoptosis/decrease proliferation). Additionally, in decidual cells-pretreated with MG-132, a proteasome inhibitor, H_2_O_2_ treatment further reduces MG-132 inhibited GRP78 levels, thereby reducing the ability of GRP78 to resolve protein-folding defects, thereby leading to prolonged ER stress as well as impaired UPR signaling in the decidua [159].

Recent in vivo observations by Wong et al. [160] demonstrate augmented ER stress in response to maternal nicotine exposure in the rat placenta accompanied by increased levels of GRP78, phosphorylated eIF2α, ATF4, and CHOP. Additional evidence indicates that a link between UPR and reduced placental protein synthesis plays key roles in the pathophysiology of FGR. Increased phosphorylation of eIF2α suggests that initiation of translation is suppressed in FGR placentas with eIF2α phosphorylation levels further increased in cases of preeclampsia complicated by FGR [161]. Furthermore, significantly elevated levels of several proteins, which are directly or indirectly associated with ER stress including GRP78, GRP94, phosphorylated (p-) PERK, eIF2a, p-eIF2a, XBP1, CHOP, IRE1, p-IRE1 and inducible nitric oxide synthase NOS expression, and reduced levels of endothelial NOS expression are observed in preeclampsia versus control placentas [162]. Reduced placental-derived placental growth factor (PlGF) levels in the maternal circulation are present in both FGR and early-onset preeclampsia. A recent study reported a correlation between reduced PlGF protein levels and nuclear localization of UPR transcription factors such as ATF4, ATF6α and ATF6β in the syncytiotrophoblast from early onset (<34 weeks) preeclamptic placentas [163]. Use of small interfering RNA-mediated mRNA degradation of ATF4 and ATF6β in BeWo cells, a trophoblast cell line, resulted in increased PlGF transcription, providing a direct evidence for suppression of PIGF by ER stress/UPR signaling [163]. Similarly, levels of ER stress markers such as PERK-induced p-eIF2α, ATF6 and XBP1u are increased in extravillous cytotrophoblasts, decidual cells and macrophages in decidual tissues derived from pregnancies complicated by FGR with or without preeclampsia [138]. Activation of placental UPR pathways including P-IRE1α, ATF6, XBP-1, GRP78 and GRP94 were all reported to be higher in early-onset (< 34 weeks) preeclampsia than in both late-onset preeclampsia and normotensive controls [164]. Although their levels are similar between second-trimester and term controls, UPR signaling (p-eIF2α, eIF2α, XBP-1 and GRP78) increases significantly in spontaneous preterm placentas delivered vaginally for acute chorioamnionitis and other conditions such as sub-chorionic and intra-parenchymal hemorrhage [164].

Impaired ER homeostasis has also been implicated in GDM e.g., increased CHOP expression in umbilical vein endothelial cells obtained from gestational diabetes mellitus, suggesting potential links between ER stress with insulin, hypercholesterolemia and/or angiogenesis in the human feto-placental vasculature [165]. A recent study provides evidence that metabolic acidosis rather than hyperglycemia is likely the cause of ER stress in GDM placenta [140]. Taken together, these results emphasize the diagnostic value of measuring ER stress and UPR signaling molecules as well as the important use of therapeutic molecules that rebalance placental ER homeostasis in protecting and preventing FGR, preeclampsia and gestational diabetes. Overall, results from previous studies relevant to modulation and impact of ER stress during pregnancy summarized in Table 1 that shows a consistent association of ER stress/UPR signaling cascades with normal pregnancy and labor as well as with PTB associated with pregnancy complications including FGR, preeclampsia and infection.

## 6. Conclusion

Current literature provide strong evidence that ER-stress/UPR signaling-mediated protein homeostatic mechanisms, generated in response to various extracellular and intracellular perturbations in several tissues of the female and male reproductive tract mediate a broad range of physiologic events including cell differentiation, survival (proliferation/apoptosis), migration, invasion, angiogenesis, and/or growth factor/cytokine release. Specially, in the ER, this involves interactions among several molecular “inspectors” that assess the quantity and quality of the tertiary and quaternary structures of newly synthesized proteins and dictates the fate of such proteins. Normally folded proteins are transported to Golgi bodies for further post-translational modification and/or packaging for secretion. Conversely, unfolded/misfolded proteins are transported to the ERAD protein complex for eventual degradation in proteasomes. Mounting evidence shows that ER stress and associated UPR signaling are important contributors to the normal functioning of reproductive tissues, including endometrial menstrual cycle changes, regulation of gametogenesis, development of the preimplantation embryo and placenta as well as maintenance of pregnancy and initiation of labor. On the other hand, disruption of ER homeostasis as a result of excess accumulation of unfolded/misfolded proteins due to prolonged and/or severe ER stress is involved in several pathologies that negatively impact on oogenesis and spermatogenesis, and induce endometriosis and endometrial/ovarian cancers as well as various pregnancy complications that result in preeclampsia, FGR and/or PTB. We posit that further research into reproductive ER homeostasis in relevant cell types will lead to novel treatments and preventative strategies to promote reproductive health.

## Figures and Tables

**Table 1:** Regulation and potential impact of endoplasmic reticulum (ER) stress molecules during normal and abnormal pregnancy conditions according to current literature.

ER Stress Molecules	Alteration/Sources	Action/Significance/Association	Pregnancy Stage/Groups
HSC70	Increased secretion from blastocyst	Paracrine action for proper folding of newly translated and misfolded proteins in decidual cells	Implantation window [133]
GRP78	Increased in endometrial stromal cells	Recurrent miscarriage	Implantation window [114]
IRE1α	Knock-out mouse Smaller placenta and embryo sizes	A reduced VEGF-A levels in the placenta as well as severe dysfunction of the labyrinth placenta	Placentation in mouse [157]
GRP78 and VCP	Down-regulation in decidual cells	Acts with oxidative stress as cofactor for molecular induction of early pregnancy loss	Specimens from Early pregnancy loss [159]
GRP78, P-eIF2α and XBP-1	Increased levels in syncytiotrophoblasts	Increased ER stress during normal labor	Labor vs. Non-labor placentas [154]
GRP78, IRE1 and XBP-1s	-In fetal membranes and myometrium -LPS mediated increase in explant cultures of fetal membranes and myometrium	- Increased ER stress in preterm and term labor - Infection may induce ER stress	Term and spontaneous pre-term labor vs. non-labor placenta specimens. Fetal membranes and myometrium from non-laboring women at the time of term Cesarean section [155]
GRP78, P-eIF2α, ATF4, and CHOP	Increased in placenta	Elevated ER stress and deregulation of proper protein folding during pregnancy	During pregnancy in rat [160]
P-eIF2α	Increased in placenta	Increased ER stress that reduce placental protein synthesis	FGR (GA weeks 28–38) vs. term control (GA weeks 39–40) [161]
GRP78 and 94, P-PERK, eIF2a, P-eIF2a, XBP1, CHOP, IRE1, P-IRE1	Elevated levels in placentas	Exaggerated ER stress in preeclampsia	Preeclamptic (mean GA weeks 33.6) vs. control placentas (mean GA weeks 39.2) [162]
UPR transcription factors ATF4, ATF6α and ATF6β	Increased nuclear localization in the syncytiotrophoblasts	Increased ER stress and contributes to reduced PlGF protein levels	Preeclamptic placentas (GA < 34 weeks ) vs. term control [163]
PERK-induced p-eIF2α, ATF6 and XBP1u	Increased levels in extra-villous trophoblasts, decidual cells and macrophages	Increased ER stress may impair placental growth associated with FGR and FGR + pre-eclampsia	Decidual tissues from FGR (mean GA weeks 31.9) or FGR with pre-eclampsia (mean GA weeks 30.3) vs. term control (mean GA weeks 38.7) [138]
P-IRE1α, ATF6, XBP-1, GRP78 and GRP94	Increased in placental lysates	Impaired ER stress may cause placental dysfunction that triggers preeclampsia	Early-onset (<34 weeks) pre-eclampsia vs. late-onset pre-eclampsia and normotensive controls [164]
P-eIF2α, eIF2α, XBP-1 and GRP78	Increased in placental lysates	Association between increased ER stress and preterm labor	Spontaneous pre-term placentas (due to acute chorioamnionitis and other conditions) vs. term controls [164]

## Abbreviations

AN3CA	An endometrial adenocarcinoma cell line
ATF6	Activating transcription factor 6
ATF6N	Activated transcription factor
Ca^2+^	Calcium
CHOP	C/EBP homologous protein
DNA	Deoxyribonucleic acid
DRP1	Dynamin-related protein 1
**E_2_**	Estradiol
EIF2AK3	Eukaryotic translation initiation factor 2-α kinase 3
EMT	Epithelial-mesenchymal transition
ER	Endoplasmic reticulum
ERAD	ER-associated degradation
FGR	Fetal growth restriction
GADD34	Growth arrest and DNA damage-inducible protein 34
GRP78	Glucose-regulated protein 78
GRP94	Glucose-regulated protein 94
H2O2	Hydrogen peroxide
HSPA5	Heat shock 70 protein 5
HeLa	A cervical adenocarcinoma cell line
ICSI	Intra-cytoplasmic sperm injection
IL-3	Interleukin-3
IL-8	Interleukin-8
IL-6	Interleukin-6
IRE1	Inositol-requiring enzyme 1
LPS	Lipopolysaccharide
MAP1LC3B	Microtubule-associated proteins 1A/1B light chain 3B
MDM2	Mouse double minute 2 homolog
MET	Mesenchymal-epithelial transition
mRNA	Messenger RNA
mtDNA	Mitochondrial DNA
NOS	Nitric oxide synthase
CDKN1A	Cyclin-dependent kinase inhibitor 1
P-ATF4	Phosphorylated-activating transcription factor 4
PDI	Protein disulfide isomerase
P-eIF2α	Phosphorylated-eukaryotic initiation factor 2α
PERK	Phosphorylated endoplasmic reticulum kinase
P-JNK	Phosphorylated-c-Jun NH2-terminal kinases
PKR	Protein kinase R
PPI	Peptidyl-prolyl isomerase
ROS	Reactive oxygen species
S1P	Site-1 protease
S2P	Site-2 protease
TFAM	Mitochondrial transcription factor A
TNFα	Tumor necrosis factor α
TUDCA	Tauroursodeoxycholic acid
UPR	Unfolded protein response
VCP	Valosin-containing protein
VEGF	Vascular endothelial growth factor
XBP1	X-box binding protein 1
